# A kidney-brain neural circuit drives progressive kidney damage and heart failure

**DOI:** 10.1038/s41392-023-01402-x

**Published:** 2023-05-12

**Authors:** Wei Cao, Zhichen Yang, Xiaoting Liu, Siqiang Ren, Huanjuan Su, Bihui Yang, Youhua Liu, Christopher S. Wilcox, Fan Fan Hou

**Affiliations:** 1 Division of Nephrology, Nanfang Hospital, Southern Medical University, State Key Laboratory of Organ Failure Research, National Clinical Research Center of Kidney Disease, Guangdong Provincial Institute of Nephrology, Guangzhou, PR China; 2 Guangdong-Hong Kong-Macao Greater Bay Area Center for Brain Science and Brain-Inspired Intelligence; Key Laboratory of Mental Health of the Ministry of Education; Guangdong Province Key Laboratory of Psychiatric Disorders, Southern Medical University, Guangzhou, Guangdong China; 3 Division of Nephrology and Hypertension, Georgetown University Medical Central, Washington, DC USA

**Keywords:** Kidney diseases, Cardiovascular diseases

## Abstract

Chronic kidney disease (CKD) and heart failure (HF) are highly prevalent, aggravate each other, and account for substantial mortality. However, the mechanisms underlying cardiorenal interaction and the role of kidney afferent nerves and their precise central pathway remain limited. Here, we combined virus tracing techniques with optogenetic techniques to map a polysynaptic central pathway linking kidney afferent nerves to subfornical organ (SFO) and thereby to paraventricular nucleus (PVN) and rostral ventrolateral medulla that modulates sympathetic outflow. This kidney-brain neural circuit was overactivated in mouse models of CKD or HF and subsequently enhanced the sympathetic discharge to both the kidney and the heart in each model. Interruption of the pathway by kidney deafferentation, selective deletion of angiotensin II type 1a receptor (AT1a) in SFO, or optogenetic silence of the kidney-SFO or SFO-PVN projection decreased the sympathetic discharge and lessened structural damage and dysfunction of both kidney and heart in models of CKD and HF. Thus, kidney afferent nerves activate a kidney-brain neural circuit in CKD and HF that drives the sympathetic nervous system to accelerate disease progression in both organs. These results demonstrate the crucial role of kidney afferent nerves and their central connections in engaging cardiorenal interactions under both physiological and disease conditions. This suggests novel therapies for CKD or HF targeting this kidney-brain neural circuit.

## Introduction

Chronic kidney disease (CKD) and heart failure (HF) contribute greatly to the current burden of disease.^1,2^ Both conditions occur with increased prevalence in the aged population, and those with hypertension, diabetes, or other cardiovascular risk factors.^3,4^ The observation that the presence of one of the conditions accelerates the progression of the other,^5,6^ suggests an important pathophysiologic link between the kidney and heart.^7^ The concept of a ‘cardiorenal syndrome’ has been proposed to encompass this important interaction^8^ but its underlying mechanisms remain poorly understood.

Several hemodynamic and neurohumoral mechanisms have been implicated in the progression of CKD or HF.^9,10^ A recent review emphasizes the important role of neural connections in cardio-renal syndrome.^11^ Whereas an overactivation of the sympathetic nervous system (SNS) can compensate for cardiovascular insults, over time, this results in cardiac dysfunction and an inability to maintain cardiac output.^12,13^ The kidney is important both for the regulation of the central sympathetic outflow^14–16^ and as an organ for driving damaging effects of efferent sympathetic outflow from the brain.^11,17–19^ The kidney has a rich sensory (afferent) innervation that we have reported drives a reflex activation of the efferent sympathetic nerves in a rat model of CKD to accelerate the progression of kidney fibrosis.^20^ Recent studies in HF report that selective interruption of kidney afferent nerves reduces lumbar SNS activity,^21^ while interruption of both kidney afferent and sympathetic efferent nerves decreases cardiac SNS activity^22^ and improves cardiac function.^23–25^ These findings suggest a hypothesis that kidney sensory (afferent) nerves relay information to the central nervous system to modulate sympathetic outflow to both the heart and the kidney. Initial animal studies suggest that many regions in the brain stem and the forebrain are activated under kidney afferent nerve stimulation.^17,26,27^ However, the precise central pathway responsive to kidney afferent nerves that control the sympathetic outflow has not been mapped, and the mechanisms underlying the activation of this central pathway remain to be elucidated.

Here we report a study that has combined virus tracing and optogenetic techniques to identify a polysynaptic central neuronal pathway that links activated kidney sensory nerves to neurons in a subfornical organ (SFO)-paraventricular nucleus (PVN)-rostral ventrolateral medulla (RVLM) pathway to control the sympathetic outflow. We further demonstrate that this kidney-brain neural circuit is activated in both experimentally induced CKD and HF and drives the SNS to accelerate disease progression in both organs.

## Results

### Kidney afferent nerves drive a kidney-to-brain neural circuit in normal mice

**Fig. 1:**
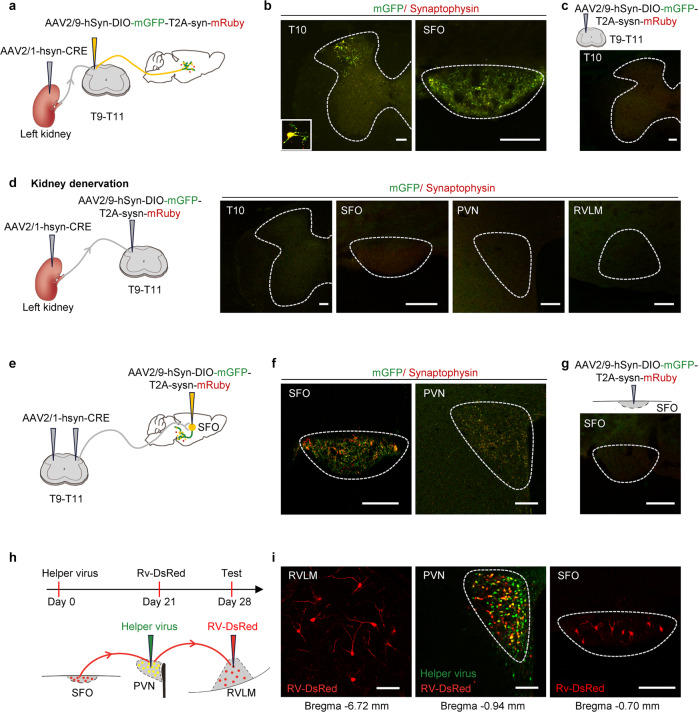
Kidney afferent nerves drive a kidney-to-brain neural circuit in normal mice. **a**, **b** Schematic for labeling presynaptic terminals of dorsal horn neurons that receive kidney afferent input **a**. Representative image shows injection site in the dorsal horn at T10 level (**b**, left). The representative image shows substantial presynaptic terminals of these dorsal horn neurons in the SFO (**b**, right). Scale bar, 100 µm. **c** Schematic for injection of AAV2/9-hSyn-DIO-mGFP-T2A-sysn-mRuby into the dorsal horn at T9-T11 level alone (upper). The representative image shows no labeled neurons observed in dorsal horn after virus injection (down). Scale bar, 100 µm. **d** Normal mice were subjected to kidney denervation before the virus injection (left). The representative image shows no labeling of dorsal horn neurons at T10 level, and no labeling of terminals of dorsal horn neurons in SFO, PVN, and RVLM post-virus injection in these mice (right). Scale bar, 100 µm. **e**, **f** Schematic for labeling terminals of SFO neurons that receive kidney spinal afferent input **e**. The representative image shows the injection site in the SFO (**f**, left). The representative image shows substantial presynaptic terminals of these SFO neurons in the PVN (**f**, right). Scale bar, 100 µm. **g** Schematic for injection of AAV2/9-hSyn-DIO-mGFP-T2A-sysn-mRuby into the SFO alone (upper). The representative image shows no labeled neurons observed in the SFO after virus injection (down). Scale bar, 100 µm. **h**, **i** Schematic for labeling direct innervation of SFO in RVLM-projecting PVN neurons **h**. Representative images showing DsRed-labeled neurons in RVLM, double-infected relay neurons in PVN, and DsRed-labeled neurons in SFO **i**. Scale bar, 100 µm. Error bars, mean ± SD. *n* = 3 in each experiment. Abbreviations for brain regions: SFO, subfornical organ (Bregma −0.70 mm); PVN paraventricular nucleus (Bregma −0.94 mm); RVLM rostral ventrolateral medulla (Bregma −6.72 mm)

Since 92% of kidney afferent nerves are reported not to contact the brain directly,^28^ a combinatorial viral approach was selected for mapping the central connections of kidney afferent nerves.^29,30^ We first transfected the kidney of mice with adeno-associated virus encoding Cre-recombinase under the control of the neuronal specific synaptophysin-promoter, AAV2/1-hSyn-CRE-WPRE-hGH pA. This is a monosynaptic, anterogradely transported virus carrying a Cre construct^29,31,32^ that was used to anterogradely transport Cre recombinase into the sensory dorsal horn of the spinal cord at T9-T11 level. This is the first relay for kidney afferent input to the brain.^14^ Next, we injected a Cre-inducible virus encoding mGFP and synaptophysin-mRuby (AAV2/9-hSyn-DIO-mGFP-T2A-sysn-mRuby-WPRE-hGH pA) into the dorsal horn at T9-T11 level to label specifically the axon presynaptic terminals of dorsal horn neurons that received direct kidney input (Fig. 1a). As shown in Fig. 1b, we found substantial presynaptic terminals of these dorsal horn neurons in the SFO. These results provide direct evidence that a subgroup of kidney afferent nerves synapse onto dorsal horn neurons that project to the SFO. A detailed description of the labeling patterns in the brain post-injection is provided in Supplementary Fig. 1a, b. By contrast, no labeled neurons were observed in the dorsal horn with injection of AAV2/9-hSyn-DIO-mGFP-T2A-sysn-mRuby-WPRE-hGH pA alone into the dorsal horn (Fig. 1c). In addition, no labeled terminals were found in brain regions from normal mice with bilaterally denervated kidneys (Fig. 1d). Thus, neuronal labeling could not be attributed to leakage of virus into the circulation and non-specific infection.

To identify the projecting targets of the SFO neurons innervated by kidney spinal afferents, the T9-T11 dorsal root entry zone of male mice was transfected with AAV2/1-hsyn-CRE-WPRE-hGH pA, while the SFO of these mice was injected with AAV2/9-hSyn-DIO-mGFP-T2A-sysn-mRuby-WPRE-hGH pA (Fig. 1e). As shown in Fig. 1f, we found substantial presynaptic terminals of these SFO neurons in the PVN. A detailed description of the labeling patterns in the brain post-injection is provided in Supplementary Fig. 1c, d. Thus, SFO neurons that receive kidney spinal afferent input project to the PVN. No labeled neurons were observed in the SFO with injection of AAV2/9-hSyn-DIO-mGFP-T2A-sysn-mRuby-WPRE-hGH pA alone into the SFO (Fig. 1g).

Since the PVN can project to the RVLM,^33^ we employed a trans-synaptic tracing method based on a modified rabies virus (RV)^29^ to determine whether RVLM-projecting PVN neurons receive direct innervation from the SFO (Fig. 1h). First, PVN neurons were infected with a Helper virus that is required for RV replication. Thereafter, we injected RV-EVNA-ΔG-DsRed into the RVLM to retrogradely infect Helper + RVLM-projecting PVN neurons. The double-infected relay neurons in the PVN produced infectious RV-DsRed that propagated transneuronally to infect SFO neurons that formed synapses with them. We observed a population of neurons in the SFO that were labeled retrogradely with RV-DsRed (Fig. 1i), suggesting direct innervation of SFO neurons in the PVN-RVLM pathway.

We conclude that the SFO-PVN-RVLM form a specific polysynaptic central pathway that normally links kidney afferent nerves to the sympathetic outflow center in mice.

### Activation of the SFO-PVN-RVLM pathway by kidney afferent nerves enhances sympathetic discharge and promotes kidney and cardiac dysfunction in models of CKD and HF

**Fig. 2:**
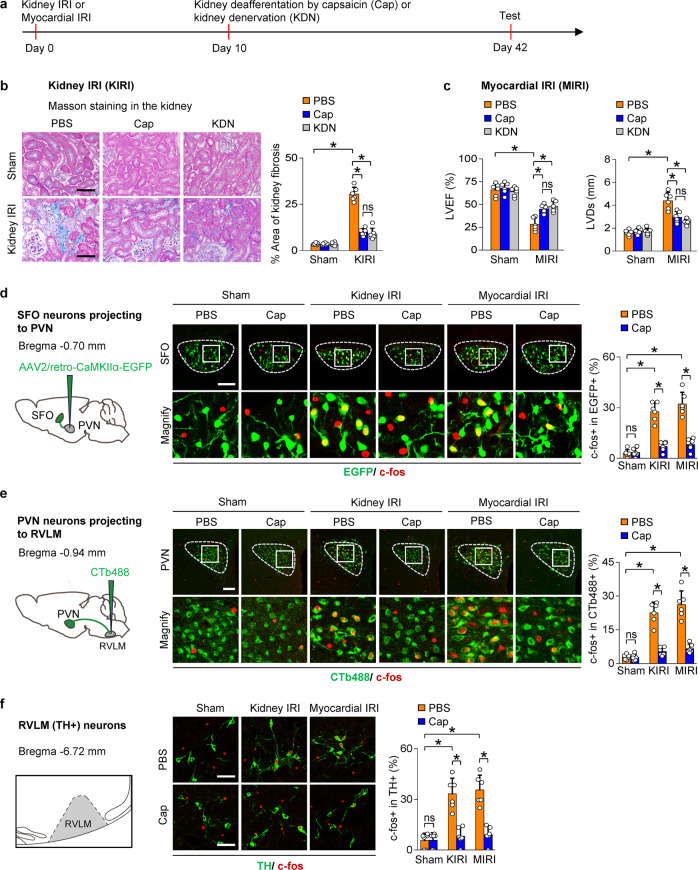
Increase in kidney afferent input drives SFO-PVN-RVLM pathway to promotes progressive kidney and cardiac dysfunction after injury. **a** Experimental models of CKD are induced by kidney IRI (KIRI), while experimental models of HF are induced by myocardial IRI (MIRI). Selective kidney deafferentation by capsaicin (Cap) or surgical ablation of kidney nerves (KDN) was performed on day 10 after KIRI or MIRI. **b** Kidney fibrosis determined by Masson staining in KIRI mice: representative images and quantitative data. Scale bar, 100 µm. **c** Left ventricular ejection fraction (LVEF, left) and LV systolic dimension (LVDs, right) in MIRI mice. **d** Retrograde labeling of SFO neurons projecting to PVN using AAV2/retro-CaMKIIα-EGFP (left). C-fos expression in EGFP-labelled SFO neurons (right): representative images and percentage of c-fos+ cells in EGFP + cells. Scale bar, 100 µm. **e** Retrograde labeling of PVN neurons projecting to RVLM using CTb-488 (left). C-fos expression in CTb-488-labelled PVN neurons(right): representative images and percentage of c-fos+ cells in CTb-488+ cells. Scale bar, 100 µm. **f** Schematic of RVLM (left). Immunostaining of c-fos and TH in RVLM (right): representative images and percentage of c-fos+ cells in TH + cells. scale bar, 50 µm. ns, not significant. *, *P* < 0.001. Error bars, mean ± SD (*n* = 6 in each group). One-way ANOVA or *t* test with Bonferroni correction

To assess the activity of the central pathways under pathological condition, we first prepared experimental model of CKD by kidney IRI and model of HF by myocardial IRI (Fig. 2a). Reduced glomerular filtration rate and increased kidney fibrosis were observed 6 weeks post kidney IRI (Fig. 2b, Supplementary Fig. 2b–d). Increased myocardial fibrosis, reduced left ventricular ejection fraction (LVEF) and enhanced LV dilatation were observed 6 weeks after myocardial IRI (Fig. 2c, Supplementary Fig. 2e, f). The HF mice also exhibited an elevated central venous pressure, a decrease in kidney vascular density, and an increase in Ang II and proinflammatory cytokines in kidney homogenates (Supplementary Fig. 2g–j). Blockade of sympathetic traffic by selective kidney deafferentation or surgical ablation of kidney nerves both lessened all these organ fibrosis and dysfunction in models of CKD and HF (Fig. 2a–c, Supplementary Fig. 2b–j). Specifically, kidney deafferentation lessened the kidney fibrosis by 67% (Fig. 2b) and improved the GFR by 41% (Supplementary Fig. 2d) in CKD model. In HF model, kidney deafferentation improved myocardial fibrosis by 71% (Supplementary Fig. 2f) and increased the LVEF by 60% (Fig. 2c). Notably, all these protective effects in the kidney and heart provided by kidney deafferentation were identical to kidney denervation in both magnitude and temporal profile (Fig. 2a–c, Supplementary Fig. 2b–j).

Virus techniques were used to label the central pathway. Since only glutaminergic SFO neurons project to the PVN,^34^ these SFO neurons were labeled by injecting a retrograde transport virus with CamKIIα-promoter (AAV2/retro-CaMKIIα-EGFP) into PVN (Fig. 2d), while PVN neurons projecting to the RVLM were labeled by injecting a retrograde tracer CTb-488 into RVLM (Fig. 2e). The virus injection site in PVN or RVLM was demonstrated in Supplementary Fig. 2k or 2l, respectively. There was robust activation of SFO neurons projecting to the PVN (Fig. 2d) and PVN neurons projecting to the RVLM in both kidney and myocardial IRI models (Fig. 2e). Consistently, activation of tyrosine hydroxylase (TH)-positive presympathetic neurons^33^ that control spinal sympathetic activity was observed in the RVLM in mouse models of both CKD and HF (Fig. 2f).

**Fig. 3:**
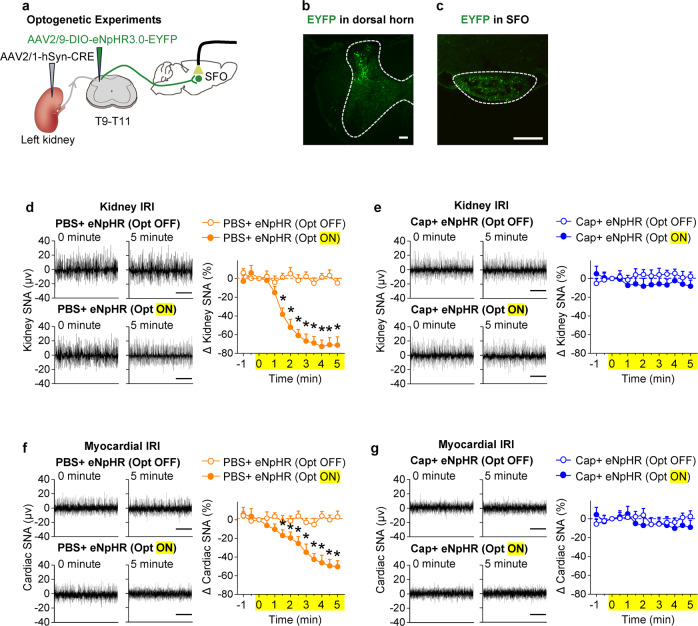
Increase in kidney afferent input drives SFO-PVN-RVLM pathway to enhance sympathetic discharge in CKD and HF. **a** Experimental design: we optically silenced the kidney-SFO projection in both models using third-generation Natronomonas halorhodopsin (eNpHR 3.0), a yellow-light-drivable proton pump, while simultaneously recording sympathetic nerve activity (SNA) in the kidney or heart. **b** Representative image of injection site in the dorsal horn at the T10 level. Scale bar, 100 µm. **c** Representative image of EYFP-positive dorsal horn neuronal terminals in the SFO. Scale bar, 100 µm. **d** Changes of kidney SNA in KIRI mice treated with PBS, with (Opt ON) or without (Opt OFF) optical silence. **e** Changes of kidney SNA in KIRI mice treated with Cap, in condition of Opt ON or Opt OFF. **f** Changes of cardiac SNA in MIRI mice treated with PBS, in condition of Opt ON or Opt OFF. **g** Changes of cardiac SNA in MIRI mice treated with Cap, in condition of Opt ON or Opt OFF. Scale bar, 2 s in **d**–**g**. The value at 0 min was set to zero on the ordinate, and changes from zero were shown as % changes in sympathetic nerve activity. *, *P* < 0.05 *versus* Opt OFF. ns, not significant. Error bars, mean ± SD (*n* = 6 in each group). One-way ANOVA or *t* test with Bonferroni correction

To test whether kidney afferent nerve signals are required to activate the SFO-PVN-RVLM pathway and sympathetic outflow in CKD or HF, selective kidney deafferentation was undertaken in the kidney and myocardial IRI models. Deafferentation of the kidneys decreased significantly the excitatory effects induced by kidney or myocardial IRI (Fig. 2d–f). Furthermore, we optically silenced the kidney-SFO projection in both models using third-generation Natronomonas halorhodopsin (eNpHR 3.0), a yellow-light-drivable proton pump, while simultaneously recording sympathetic nerve activity (SNA) in the kidney or heart. First, we labeled the dorsal horn neurons that receive kidney input by delivering an AAV2/1-hSyn-CRE-WPRE-hGH pA into the kidney and an AAV2/9-EF1α-DIO-eNpHR3.0-EYFP-WPRE-hGH pA into the dorsal horn at the T9-T11 level (Fig. 3a, b). Ascending EYFP-positive terminals of dorsal horn neurons that receive direct kidney input were concentrated in the SFO (Fig. 3c). Thereafter, an optic fiber was placed immediately above these neuronal terminals in the SFO to deliver the yellow laser light through the fiber. This markedly reduced the sympathetic discharge in both myocardial and kidney IRI models (Fig. 3d, f), while kidney deafferentation abolished the sympathetic response to optical silence (Fig. 3e, g), thereby demonstrating its dependence on kidney afferent nerves.

Interestingly, the increased sympathetic outflow that was induced by kidney IRI was observed not only in the kidney but also in the heart of the CKD model (Supplementary Fig. 3a). Similarly, the increase in sympathetic discharge induced by myocardial IRI was observed not only in the heart but also in the kidney (Supplementary Fig. 3b). We conclude that both experimental HF and experimental CKD activate kidney afferent nerves that drive SFO-PVN-RVLM connections to provide a pathway that links a damaged kidney to the heart or a damaged heart to the kidney.

### Activation of SFO-PVN-RVLM pathway in CKD and HF depends on activation of renin-angiotensin system (RAS) in SFO

**Fig. 4:**
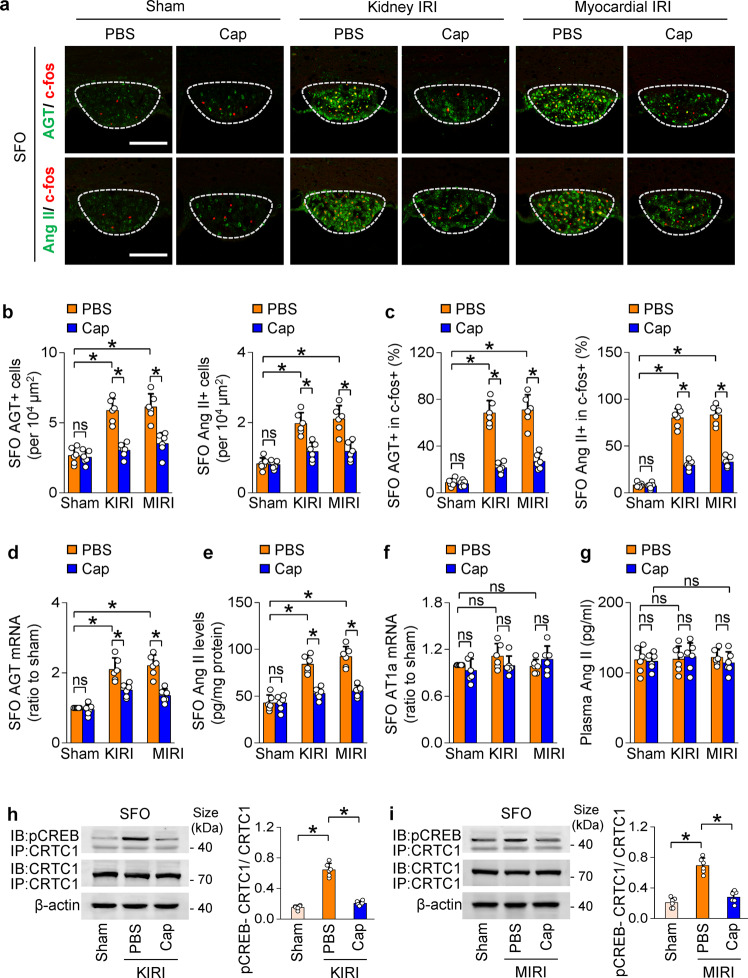
Kidney afferent inflow in CKD and HF activates RAS in SFO. **a** Immunostaining of c-fos with AGT or Ang II in SFO of kidney IRI (KIRI) or myocardial IRI (MIRI) mice. Scale bar, 100 µm. **b** Quantitative analysis of AGT + (left) and Ang II + (right) cells in SFO. **c** Percentage of AGT + (left) or Ang II + (right) cells in c-fos+ cells in SFO. **d**–**f** Level of AGT mRNA **d**, Ang II protein **e**, and AT1a mRNA **f** in homogenates of SFO from KIRI or MIRI mice. **g** Plasma Ang II level in KIRI or MIRI mice. **h**, **i** Binding of CRTC1 with phosphorylated CREB in SFO of KIRI **h** or MIRI **i** mice. ns not significant. *, *P* < 0.001. Error bars, mean ± SD (*n* = 6 in each group). One-way ANOVA or *t* test with Bonferroni correction

The demonstration in rodents of strong expression of angiotensin II type 1a (AT1a) and AT1b in the SFO suggests that it is a major site for transduction of Ang II to neural signals.^35,36^ As shown in Fig. 4a–e, the expression of AGT and Ang II were upregulated in activated neurons (marked by c-fos) in the SFO of both myocardial and kidney IRI mice. The expression of AT1a in the SFO and the levels of Ang II in the plasma were unchanged in mice with kidney and heart damage (Fig. 4f, g). Importantly, kidney deafferentation reduced the experimental upregulation of AGT and Ang II in the SFO significantly in both myocardial and kidney IRI mice (Fig. 4a–e).

To explore how kidney afferent signals trigger the local RAS activation in HF and CKD, AGT gene transcription in the SFO was assessed by determining dephosphorylation of CRTC1 that is a transcriptional coactivator of nuclear transcriptional factor CREB for AGT,^37,38^ and the binding of active CRTC1 to phosphorylated CREB. Dephosphorylation of CRTC1 and binding of CRTC1 with phosphorylated CREB in the SFO were significantly increased in both kidney and heart models and were prevented by kidney deafferentation (Fig. 4h, i; Supplementary Fig. 4).

**Fig. 5:**
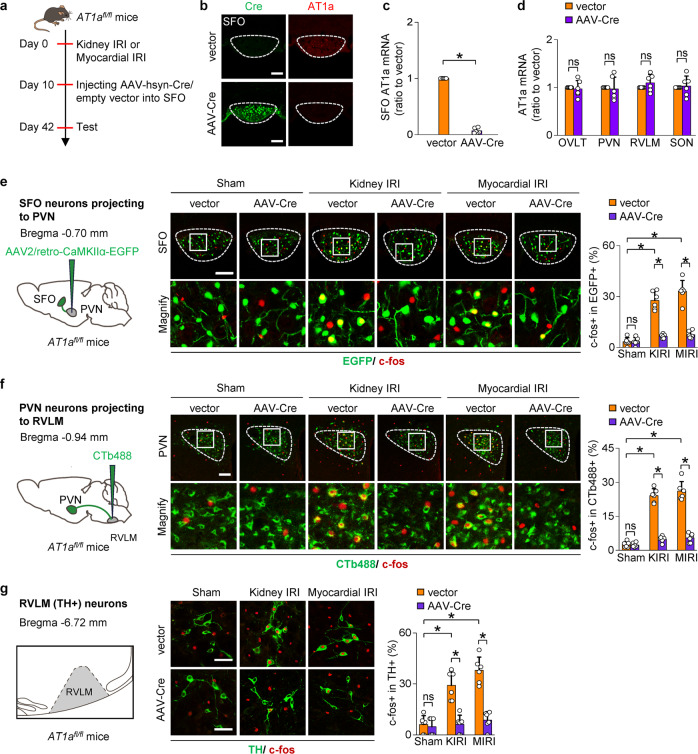
Activation of SFO-PVN-RVLM pathway in CKD and HF depends on activation of RAS in SFO. **a** Deletion of AT1a in SFO is achieved by injection of AAV2/9-Cre into SFO of AT1a^*fl/fl*^ mice on day 10 after kidney IRI (KIRI) or myocardial IRI (MIRI). **b**–**d** Representative images show immunostaining of Cre and in situ hybridization of AT1a mRNA in the SFO of AT1a^*fl/fl*^ mouse treated with AAV-Cre or empty vector **b**. Scale bar, 100 µm. The mRNA expression of AT1a is determined in homogenates of SFO **c**, OVLT, PVN, RVLM, and SON **d**. **e** Retrograde labeling of SFO neurons projecting to PVN using AAV2/retro-CaMKIIα-EGFP. C-fos expression in EGFP-labelled SFO neurons: representative images and percentage of c-fos+ cells in EGFP + cells. Scale bar, 100 µm. **f** Retrograde labeling of PVN neurons projecting to RVLM using CTb-488. C-fos expression in CTb-488-labelled PVN neurons: representative images and percentage of c-fos+ cells in CTb-488+ cells. Scale bar, 100 µm. **g** Schematic of RVLM. Immunostaining of c-fos and TH in RVLM: representative images and percentage of c-fos+ cells in TH + cells. Scale bar, 50 µm. *P < 0.001. ns, not significant. Error bars, mean ± SD (*n* = 6 in each group). One-way ANOVA or *t* test with Bonferroni correction

To determine the role of RAS activation in triggering the SFO-PVN-RVLM pathway, mice were generated with specific deletion of AT1a in the SFO by injection of AAV2/9-hsyn-CRE-WPRE-hGH pA into SFO (Fig. 5a). The expression of AT1a in SFO was determined by in situ hybridization and real-time PCR. The mRNA of AT1a almost disappeared from the SFO of AT1a-gene deleted mice (Fig. 5b, c). This effect was specific for the SFO since the levels of AT1a mRNA were maintained in the nearby nuclei (Fig. 5d).

**Fig. 6:**
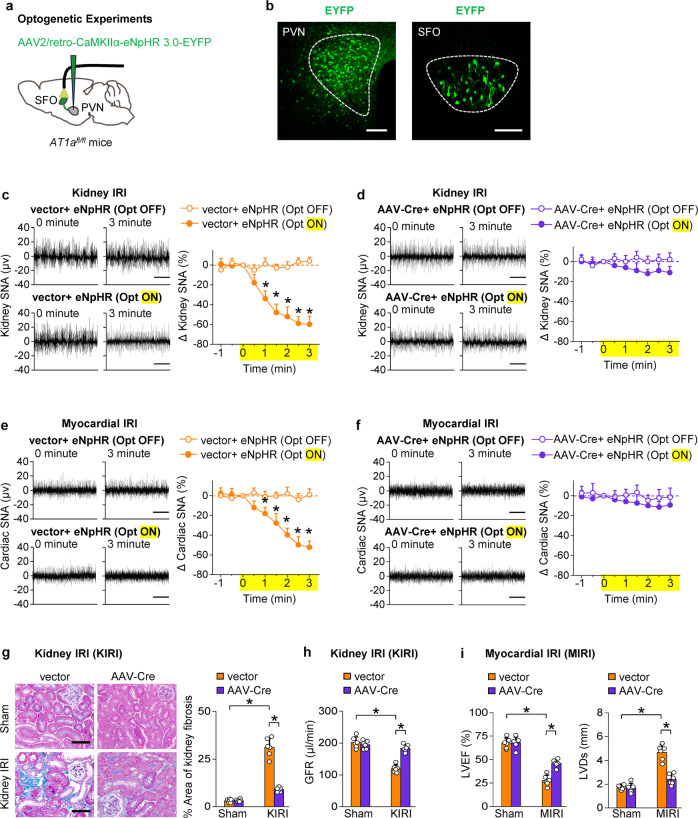
Activation of the SFO RAS enhance sympathetic discharge and promotes progressive kidney and cardiac dysfunction after injury. **a** Schematic showing optogenetic silence of SFO neurons projecting to PVN with simultaneous recording of sympathetic nerve activity (SNA) in the kidney or heart. **b** Representative image of injection site in the PVN (left), and representative image of EYFP-positive neurons in SFO (right). Scale bar, 100 µm. **c** Changes of kidney SNA in KIRI mice treated with empty vector, in condition of Opt ON or Opt OFF. *P < 0.05 *versus* Opt OFF. **d** Changes of kidney SNA in AT1a-deleted KIRI mice, in condition of Opt ON or Opt OFF. **e** Changes of cardiac SNA in MIRI mice treated with empty vector, in condition of Opt ON or Opt OFF. *P < 0.05 *versus* Opt OFF. **f** Changes of cardiac SNA in AT1a-deleted MIRI mice, in condition of Opt ON or Opt OFF. **g** Kidney fibrosis determined by Masson staining in KIRI mice: representative images (scale bar, 100 µm) and quantitative data. *, *P* < 0.001. **h** GFR in KIRI mice. **i** Left ventricular ejection fraction (LVEF, left) and LV systolic dimension (LVDs, right) in MIRI mice. Scale bar in **c**–**f**, 2 s. The value at 0 min is set to zero in **c**–**f**. *, *P* < 0.001. ns, not significant. Error bars, mean ± SD (*n* = 6 in each group). One-way ANOVA or *t* test with Bonferroni correction

Deleting AT1a in the SFO in myocardial or kidney IRI models reduced the activation of SFO neurons projecting to PVN, PVN neurons projecting to RVLM and TH-positive neurons in the RVLM (Fig. 5e–g). Moreover, to examine the effects of the optogenetic inhibition of SFO neurons projecting to the PVN, AAV2/retro-CaMKIIα-eNpHR 3.0-EYFP-WPRE-hGH pA was injected into the PVN of CKD or HF mice, and the cell bodies of SFO neurons with eNpHR3.0 were exposed to yellow light (Fig. 6a, b). Optical silencing of SFO neurons projecting to the PVN reduced the sympathetic outflow markedly in myocardial or kidney IRI mice (Fig. 6c, e). Deletion of AT1a in SFO abolished the sympathetic response to optical silence (Fig. 6d, f).

Consistently, deletion of AT1a in SFO lessened the organ fibrosis and dysfunction in both models of HF and CKD (Fig. 6g–i, Supplementary Fig. 5). Specifically, deletion of AT1a in SFO lessened the kidney fibrosis by 71% (Fig. 6g) and improved the GFR by 53% (Fig. 6h) in CKD model. In HF model, deletion of AT1a in SFO improved myocardial fibrosis by 74% (Supplementary Fig. 5c) and increased the LVEF by 66% (Fig. 6i). Thus, activation of a local RAS by kidney afferents in the SFO is required to drive the central neural pathway in myocardial and kidney IRI mouse models.

## Discussion

**Fig. 7:**
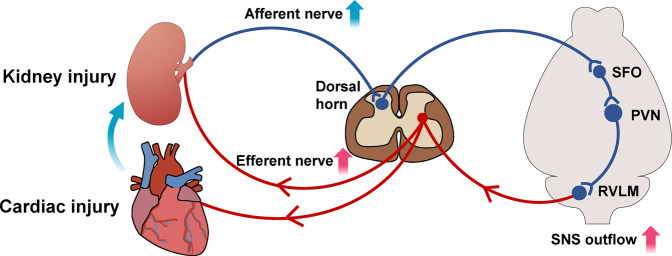
Schematic diagram summarizing a polysynaptic central neuronal pathway that links activated kidney sensory nerves to neurons in SFO-PVN-RVLM pathway to control the sympathetic outflow. This kidney-brain neural circuit is overactivated in both experimentally induced CKD and HF, and drives the sympathetic nervous system (SNS) to accelerate disease progression in both organs. [created with Adobe Illustrator (Adobe, San Jose, CA)]

We have mapped a polysynaptic central pathway composed of SFO-PVN-RVLM neurons that links kidney afferent nerves to activation of the RVLM and thereby the sympathetic outflow (Fig. 7). This kidney-brain neural circuit becomes overactive in mouse models of either CKD or HF and mediates an enhanced sympathetic discharge to both the heart and the kidney in each model. The pathway is driven by kidney afferent nerves and its function depends on RAS activity in SFO, since it is inhibited by kidney deafferentation or selective deletion of AT1a in SFO. Both interruptions decrease sympathetic discharge and lessen structural abnormality and dysfunction in the hearts and kidneys of mice with experimentally induced CKD or HF. These findings provide a novel link between kidney and heart in physiological and pathological settings.

Compared with the widely studied neural pathways from the brain to the kidney,^39,40^ the precise central pathway linking kidney afferent nerve signals to sympathetic outflow remains unmapped.^11^ We use virus tracing techniques to identify neurons innervated by kidney spinal afferents that are found to congregate in the SFO. These SFO neurons project to the PVN, and eventually to the RVLM that is a gateway for controlling sympathetic outflow. The functional importance of this kidney-brain neural pathway in CKD and HF is shown by kidney deafferentation, and by experiments using optogenetic methods to silence the kidney-SFO or SFO-PVN projection, all of which abolish the increase in sympathetic discharge and lessen the dysfunction in both the heart and the kidney. This demonstrates that the kidney-brain neural circuit is driven by kidney afferent nerves and underlies cardiorenal interactions that determine the disease progression in both CKD and HF.

The kidney is heavily innervated with sensory nerves that transmit information from kidney mechano- and chemo-receptors.^41^ Although kidney ischemia,^42^ kidney dysfunction^43^ or a high-salt intake,^20^ can all stimulate afferent fibers to increase sympathetic tone,^43^ the mechanisms that activate kidney afferent nerves in HF remain obscure. Studies have highlighted the importance of kidney venous congestion,^44^ impaired kidney hemodynamics^45^ and activation of the RAS^46^ in the pathophysiology of HF. Here we demonstrate that elevated venous pressure, reduced kidney perfusion, and activation of local kidney mediators including RAS could trigger kidney afferent nerve activation in HF. Indeed, these are all maneuvers that have been demonstrated previously to increase the kidney afferent nerve activity by stimulation of intrarenal mechanoreceptor and chemoreceptors.^20,47–50^

It is interesting to discover that the activation of the SFO-PVN-RVLM pathway is dependent on RAS activity in SFO. We observe an increase in both AGT expression and Ang II levels in activated neurons of the SFO in models of HF or CKD in the absence of increases in circulating Ang II. Inflow from kidney afferent nerve fibers may trigger the activation of a local RAS in the SFO, since dephosphorylation of CRTC1 and the binding of CRTC1 with phosphorylated CREB that are critical steps in AGT transcription^37,38^ are increased in the SFO in these models and are abolished by kidney deafferentation. Furthermore, our finding that specific deletion of AT1a in the SFO blocks the activation of the central pathway, decreases the sympathetic discharge, and lessens kidney and heart dysfunction demonstrate the critical role of the local RAS in the SFO for initiating the activation of the central pathway and its pathophysiological consequences. As well-known, AT1a blockade by orally administration of AT1a antagonists have not been entirely effective in preventing sympathetic nervous and inflammatory cytokine activation. However, blockade of central AT1a by intracerebroventricular administration of AT1a antagonist losartan, at a dose of 0.2% of the effective intragastric dose, inhibited the overexpression of RAS.^20^ Similarly, since central effects of mineralocorticoid receptor (MR) activation are mediated via stimulation of angiotensinergic sympathoexcitatory pathways,^51,52^ central MR blockade might be of therapeutic benefit for patients with CKD and HF.

A comprehensive understanding of the mechanisms will enhance our ability to treat cardiorenal conditions and their cardiovascular complications more efficaciously and thoroughly.^11^ Catheter-based kidney denervation has been introduced to treat drug-resistant hypertension,^53,54^ but is now proposed to treat HF.^55,56^ The major beneficial effects of kidney denervation are thought to depend on removal of the kidney efferent nerve fibers,^57,58^ but an important role of kidney deafferentation is suggested by our study. Thus, in both models of CKD and HF, an increased afferent nerve inflow is shown to drive the activation of the central pathway, resulting in increased sympathetic discharge. Many reports have demonstrated that the increased sympathetic discharge in the kidney promotes inflammation, oxidative stress, activation of RAS, and accelerates kidney fibrosis.^14,19^ Moreover, ablation of kidney afferent nerves reduces sympathetic outflow to both kidney and heart significantly in each model of CKD and HF. This suggests that protection of both kidney and heart by kidney nerve ablation might be ascribed in part at least to kidney deafferentation.

Our findings have other potential clinical implication that could be explored in targeted studies. First, the SFO-PVN-RVLM pathway could become a therapeutic target potentially accessible to transcranial inhibition, to prevent progressive cardiac or kidney dysfunction after injury. Second, the finding that activation of a local RAS in the SFO is required for promoting progression of cardiac and kidney dysfunction after injury, identifies a potential central site of action for drugs that inhibit RAS in addition to their well-established kidney and cardiac sites.

In conclusion, we identify a kidney to brain neural circuit that is driven by kidney afferent nerves linked to efferent sympathetic outflow. This kidney-brain neural circuit is activated in experimentally induced CKD or HF and contributes to progression of kidney and cardiac dysfunction. These results provide novel understanding of how kidney afferent nerves may provide a previously underappreciated mechanism contributing to the enigmatic cardiorenal syndrome. It opens further avenue of research to develop novel therapies targeting the kidney-brain neural circuit.

## Methods

Detailed methods used in this study are provided in the Data Supplement.

### Mouse models of CKD and HF

Animal procedures were approved by the Institutional Animal Ethics Committee. Surgeries were performed under anesthesia with intraperitoneal (i.p.) injection of sodium pentobarbital (50 mg/kg body weight). *Agtr1a*^*flox/flox*^ (AT1a^*fl/fl*^) mice on a C57BL/6 J background were obtained from Cyagen Bioscience (Guangzhou, China).^59^ Wild-type C57BL/6 J mice were obtained from our Institutional Animal Experiment Center. Mice aged 8 to 10 weeks (20–24 g) were used.

The CKD model of kidney ischemia-reperfusion injury (IRI) was induced by clamping both kidney pedicles for 35 min.^49^ Animals were followed up for 6 weeks. Sham-operated mice (sham) were subjected to exposure of kidneys without the induction of ischemia.

The HF model of myocardial IRI was generated by clamping of the left anterior descending coronary artery for 45 min.^60^ Sham mice underwent the identical procedure without the arterial ischemia. Animals were followed up for 6 weeks.

To block the kidney-brain neural circuit, two approaches were taken in groups of kidney or myocardial IRI mice on day 10 post surgery: total denervation of both kidneys,^42^ and selective deafferentation of both kidneys with capsaicin^42,61^ (Sigma, St. Louis, MO). The efficacy of capsaicin treatment^42^ was demonstrated by reduced kidney expression of calcitonin gene-related peptide (CGRP), an afferent nerve-specific marker (Supplementary Fig. 2a). The effectiveness of denervation^42^ was validated by reduced kidney norepinephrine levels (normal kidney *versus* denervated kidney: 0.28 ± 0.05 versus 0.03 ± 0.01 ng/mg protein).

### Brain surgery and viral injection

Mice were anesthetized by sodium pentobarbital (50 mg/kg, i.p.) and placed in a stereotactic frame (Stoelting, IL, USA). A craniotomy hole was drilled, and the virus was injected via a glass micropipette connected to a Nanoliter Inject (NANOLITER 2020, WPI, FL, USA) and its controller (Micro4, WPI) at a speed of 100 nL/min. Injection coordinates were respect to bregma, according to the *Paxios and Franklin Mouse Brain Atlas*, third edition. All viruses were from BrainVTA.

### SFO angiotensin II type 1a receptor-deleted mouse

To achieve SFO-specific deletion of angiotensin II type 1a receptor (AT1a), the SFO of an AT1a^*fl/fl*^ mouse was transfected with adeno-associated virus encoding Cre-recombinase under the control of the neuronal specific synaptophysin-promoter as previously described^62^ (AAV2/9-hsyn-CRE-WPRE-hGH pA, BrainVTA, Wuhan, China; at a speed of 100 nL/min for 3 min, coordinate relative to bregma: anteroposterior, −0.6 mm; lateral, ±0.0 mm; ventral, +2.5 mm).

### Anatomical tracing studies

#### Anterograde tracing

To identify the nuclei that receive direct innervation from dorsal horn neurons that receive kidney afferent input, AAV2/1-hSyn-CRE-WPRE-hGH pA was injected into the left kidney (Virus titers: 1.0 × 10^13^ vector genomes/mL, 4 µL/kidney), and AAV2/9-hSyn-DIO-mGFP-T2A-sysn-mRuby-WPRE-hGH pA, which allows for analysis of Cre-inducible synaptophysin expression was injected bilaterally into T9-T11 dorsal root entry zones (Virus titers: 5.0 × 10^12^ vector genomes/mL, 0.3 µL/dorsal horn; coordinates: 0.4 mm from the midline, 0.4 mm deep).

To delineate the targets of SFO neurons innervated by kidney spinal afferents, AAV2/1-hSyn-CRE-WPRE-hGH pA was injected bilaterally into T9-T11 dorsal root entry zones (0.3 µL/injection), and AAV2/9- hSyn-DIO-mGFP-T2A-sysn-mRuby-WPRE-hGH pA was injected into the SFO (0.3 µL^62^).

#### Retrograde tracing

For di-synaptic tracing of the SFO-PVN-RVLM pathway, Helper virus (AAV2/9-CaMKIIα-EGFP-T2A-TVA-WPRE-hGH pA and AAV2/9-CaMKIIα-oRVG-WPRE-hGH pA) was injected bilaterally into PVN (Virus titers: 2 × 10^12^ vector genomes/mL, 0.3 µL/PVN^33^; coordinate relative to bregma: anteroposterior, −0.7 mm; lateral, ±0.3 mm; ventral, 4.9 mm). Twenty-one days later, rabies virus (RV)-EVNA-ΔG-DsRed was injected bilaterally into the RVLM nuclei (Virus titers: 2 × 10^8^ infectious units/mL, 0.3 µL/RVLM^33^; coordinates relative to bregma: anteroposterior, -6.8 mm; lateral, ±1.1 mm; ventral, +5.9 mm).

To retrogradely label PVN-projecting SFO neurons, a retrograde AAV2/retro-CaMKIIα-EGFP was injected bilaterally into PVN (Virus titers: 2 × 10^12^ vector genomes/mL, 0.3 µL/PVN).

To retrogradely label RVLM-projecting PVN neurons, Alexa Fluor 488-conjugated Cholera Toxin Subunit B (CTB-488, Invitrogen, NY, USA) was injected bilaterally into RLVM (1 µg/µL, 0.5 µL/RVLM).^33^

All stereotaxic injection sites were verified by immunohistochemistry. Only data from correctly verified target site were included for the analyses.

### Optogenetic experiments

For optical silencing of kidney-SFO projection, AAV2/1-hSyn-CRE-WPRE-hGH pA was injected into the left kidney (Virus titers: 1 × 10^13^ vector genomes/mL, 4 µL/kidney) and the AAV2/9-EF1α-DIO-eNpHR3.0-EYFP-WPRE-hGH pA into both T9-T11 dorsal root entry zones (Virus titers: 5 × 10^12^ vector genomes/mL, 0.3 µL/dorsal horn). Thereafter, an optic fiber was placed in cannulas and inserted toward the SFO through a craniotomy to deliver laser beam from a yellow light laser (593.5 nm, 100 mW, QAXK-LASER-593.5; ThinkerTech Nanjing BioScience Inc. Nanjing, China).^33^ To achieve inhibition with eNpHR3.0, the laser output was maintained at 10–15 mW/mm^2^. The distance from the optical fiber tip to the SFO was set at 200 µm, and the expected optical power was 1.97–2.89 mW/mm^2^.

For optical silencing of PVN-projecting SFO neurons, AAV2/retro-CaMKIIα-eNpHR 3.0-EYFP-WPRE-hGH pA was injected into PVN (Virus titers: 2 × 10^12^ vector genomes/mL, 0.3 µL/PVN) and optical experiments performed as described above.

### Statistical analyses

Continuous variables were expressed as means and standard deviations. The normality of the data was tested by the Shapiro-Wilk test. Differences between 2 groups were tested by an unpaired Student *t* test. Differences among groups were tested by ANOVA or unpaired *t* test with Bonferroni correction for multiple testing. Statistical analyses were conducted with SPSS 20.0 for Windows (SPSS, Inc., Chicago, IL). A *P* value of less than 0.05 was considered statistically significant.

## Supplementary information

Supplementary Materials

## Data Availability

All data supporting the findings of this study are available from the corresponding author on reasonable request.
