# Supplementary material for: A kidney-brain neural circuit drives progressive kidney damage and heart failure
Source: Signal Transduct Target Ther. 2023 May 12;8:184. doi: 10.1038/s41392-023-01402-x (PMC10175540; doi:10.1038/s41392-023-01402-x)

**Supplementary Materials for**

**A kidney-brain neural circuit drives progressive kidney damage and heart failure**

Wei Cao,^1^ Zhichen Yang,^1^ Xiaoting Liu,^1^ Siqiang Ren,^2^ Huanjuan Su,^1^ Bihui Yang,^1^ Youhua Liu,^1^ Christopher S Wilcox,^3^ Fan Fan Hou^1^

Correspondence to: [ffhouguangzhou@163.com](mailto:ffhouguangzhou@163.com)

**This file includes:**

Methods

Figures. S1 to S5

Table. S1

Uncropped films of Western blots

**METHODS**

**Recording of sympathetic nerve activity**

Mice were anesthetized with urethane (1.0 g/kg body weight, i.p.) and placed on a controlled heating pad. For recording sympathetic nerve activity to the kidney,^1^ the nerve bundle supplying the kidney was isolated and severed. The nerves proximal to the cut were placed on a pair of platinum electrodes to record kidney sympathetic nerve activity. For recording cardiac sympathetic nerve activity,^2^ cardiac sympathetic nerve fiber was identified as a branch from the stellate ganglion and attached to a pair of platinum electrodes.

Recorded nerve signal was amplified 10000 times with an ERS 100C amplifier and filtered with a band-pass between 1 and 3000Hz.^3^ Nerve signals were analyzed on a computer and recording software (Acqknowledge, Biopac System, CA, USA) to remove background noise and count the number of spikes.^4^ The nerve activity was expressed in spikes per second (Hz), as commonly used by previous studies.^4-8^ In each animal during optogenetic experiments, nerve activity just before the optical silence was set as 0%, and percentile changes from 0% were shown as % changes in sympathetic nerve activity.

**Histology analysis**

Mice were perfused transcardially with PBS followed by fixation with 4% Paraformaldehyde.^9^ Selected tissues were harvested and dehydrated.^9,10^ Kidney or cardiac histology was assessed on paraffin sections (3 µm) stained with hematoxylin-eosin and Masson’s trichrome.^11^ Degree of tissue fibrosis was quantitated by assessing the percentage of fibrosis in each section.^11^

**Evaluation of kidney function by glomerular filtration rate**

The glomerular filtration rate (GFR) was determined by inulin clearance.^11^ Briefly, under isoflurane anesthesia, a catheter was placed in the bladder for urine collection, and a cannula was placed in the tail vein to infuse inulin.^11^ Normal saline solution (0.9%) containing 15% inulin and 1% bovine albumin was infused at a rate of 5 µL/min.^11^ After a 1-hour equilibration period, urine was collected every 30 min for three times and blood was drawn at the end of the infusion.^11^ Inulin concentrations in urine and plasma were measured by using commercial kits (Glory Science Co., TX, USA).

**Evaluation of cardiac function by echocardiography**

Transthoracic 2-D, M-mode, and Doppler echocardiography measurements were acquired with a Vevo 2100 imaging system (VisualSonics, Toronto，Canada) on mice anesthetized with continuous respiratory inflow of isoflurane (2-3% for induction; 0.25-2% for maintenance).^12^ All M-mode tracings were recorded through the anterior and posterior LV walls at the level of the papillary muscle.^12^ Left ventricular end-systolic dimension (LVDs) was measured, and the LV ejection fraction (LVEF) was calculated using Teichholz method.

**Immunostaining**

Immunostaining was performed in paraffin tissue sections (3 µm) or frozen brain sections (30 µm). Sections were incubated with primary antibodies at 4 °C for 1 day and then reacted with secondary antibodies at 4 °C for 1 day.^9,10^ Sections were mounted on slides and images were captured on a Zeiss LSM 710 confocal (Wetzlar, Germany) or Olympus microscope (Tokyo, Japan). The primary antibodies used were listed as follows: anti-angiotensinogen (AGT) (1:100, A6279, ABclonal, China), anti-Ang II (1:800, T-4007, Peninsula Laboratories, CA, USA), anti-Cre (1:1000, ab190177, Abcam, Cambridge, UK), anti-c-fos (1:100, M00297-3), TH (1:100, PB0470) (all from Boster, Wuhan, China), anti-tdTomato (1:50, Orb182397, Biorbyt, Cambridge, UK), anti-GFP (1:100, 2207528, Invitrogen), anti-CGRP (1:100, BML-CA1134-0100, Enzo Life Sciences, Lausen, Switzerland), and anti-CD31 (1:50, AF3628, R&D Systems, Minnesota, USA) antibodies.

**In situ hybridization**

Expression of AT1a in brain sections was determined by in situ hybridization.^13^ After immunostaining with anti-Cre antibody, frozen brain sections (30 µm) were digested with proteinase K, and then hybridized with a digoxigenin-labeled LNA^TM^ Detection Probe for AT1a (Qiagen, Hilden, Germany) at 54 °C for more than 16 hours.^13^ Next, the sections were incubated with peroxidase-conjugated sheep anti-digoxigenin (1:5000, 11-207-733-910, Roche, Basel, Switzerland) at 4 °C for 16 hours, and then detected with the TSA-Plus Fluorescence System (K1051, APExBIO, TX, USA). The template used for the probe preparation was the 757-bp fragment of mouse Agtr1a (nucleotide residues 1485-2242; Genbank accession number NM_177322).^13^ The probe sequences were /5DiGN/ATGTCACGGTTGGTACAAGCA/3DiG _N/.^13^

**Measurement of Ang II concentrations**

Ang II levels in plasma or tissue homogenates were determined by liquid chromatography/mass spectrometry.^14^ Samples were extracted and separated with C18 Sep-Pak cartridges (Thermo Scientific, MA, USA), followed by detection with Q-ToF mass analyzer in the ESI+-MS ion mode.^14^

**Measurement of central venous pressure**

Under isoflurane anesthesia, the right external jugular vein was catheterized with a 22 FR polyethylene catheter (ID: 0.6 mm, OD: 0.9 mm; Terumo, Tokyo, Japan) which was connected to a water-filled polyethylene tube for measurement of the central venous pressure.^15^

**Western Blot**

Tissues were lysed and resolved by SDS-polyacrylamide gel electrophoresis.^10^ Then proteins were transferred to polyvinylidene difluoride membranes (Millipore, MA, USA), which were incubated with the following primary antibodies: anti-collagen I (1:300, BA0325, Boster), anti-CRTC1 (1:500, PA5-82786, Thermo Scientific), anti-Phospho-CREB (1:500, 9198S), anti-Phospho-CRTC1(1:500, 3359S) (all from Cell Signaling Technology). Antibody against β-actin (GTX109639, 1:1000, GeneTex, CA, USA) was applied as loading controls on stripped membranes.

**Real-Time PCR**

Total RNA was isolated from tissues with TRIzol reagent (MRC, OH, USA), and then was reverse transcribed with the reverse transcriptase Kit (Takara Biotechnology, Shiga, Japan). Real-time PCR was performed using TaKaRa SYBR® Premix Ex Taq^TM^ II kit (Takara Biotechnology). GAPDH was used as the internal control. Primers were listed in Supplementary Table 1.

**REFERENCES**

1. Niijima, A. Afferent signals from leptin sensors in the white adipose tissue of the epididymis, and their reflex effect in the rat. *J Auton Nerv Syst* **73**, 19-25 (1998).

2. Schwenke, D.O.*, et al.* One dose of ghrelin prevents the acute and sustained increase in cardiac sympathetic tone after myocardial infarction. *Endocrinology* **153**, 2436-2443 (2012).

3. Garcia, M.L.*, et al.* The antioxidant effects of green tea reduces blood pressure and sympathoexcitation in an experimental model of hypertension. *J Hypertens* **35**, 348-354 (2017).

4. Veiga, A.C.*, et al.* Selective afferent renal denervation mitigates renal and splanchnic sympathetic nerve overactivity and renal function in chronic kidney disease-induced hypertension. *J Hypertens* **38**, 765-773 (2020).

5. Ma, M.C., Huang, H.S. & Chen, C.F. Impaired renal sensory responses after unilateral ureteral obstruction in the rat. *J Am Soc Nephrol* **13**, 1008-1016 (2002).

6. Lopes, N.R.*, et al.* Afferent innervation of the ischemic kidney contributes to renal dysfunction in renovascular hypertensive rats. *Pflugers Arch* **472**, 325-334 (2020).

7. Cao, W.*, et al.* Adipocytes initiate an adipose-cerebral-peripheral sympathetic reflex to induce insulin resistance during high-fat feeding. *Clin Sci (Lond)* **133**, 1883-1899 (2019).

8. Booth, L.C.*, et al.* Renal, Cardiac, and Autonomic Effects of Catheter-Based Renal Denervation in Ovine Heart Failure. *Hypertension* **78**, 706-715 (2021).

9. Nomura, K.*, et al.* [Na(+)] Increases in Body Fluids Sensed by Central Nax Induce Sympathetically Mediated Blood Pressure Elevations via H(+)-Dependent Activation of ASIC1a. *Neuron* **101**, 60-75 e66 (2019).

10. Cao, W.*, et al.* A Salt-Induced Reno-Cerebral Reflex Activates Renin-Angiotensin Systems and Promotes CKD Progression. *J Am Soc Nephrol* **26**, 1619-1633 (2015).

11. Cao, W.*, et al.* Contrast-Enhanced Ultrasound for Assessing Renal Perfusion Impairment and Predicting Acute Kidney Injury to Chronic Kidney Disease Progression. *Antioxid Redox Signal* **27**, 1397-1411 (2017).

12. Lin, H.B.*, et al.* Innate Immune Nod1/RIP2 Signaling Is Essential for Cardiac Hypertrophy but Requires Mitochondrial Antiviral Signaling Protein for Signal Transductions and Energy Balance. *Circulation* **142**, 2240-2258 (2020).

13. Matsuda, T.*, et al.* Distinct neural mechanisms for the control of thirst and salt appetite in the subfornical organ. *Nat Neurosci* **20**, 230-241 (2017).

14. Ali, Q., Wu, Y., Nag, S. & Hussain, T. Estimation of angiotensin peptides in biological samples by LC/MS method. *Anal Methods* **6**, 215-222 (2014).

15. Shinomiya, S.*, et al.* Nitric oxide and beta(2)-adrenoceptor activation attenuate pulmonary vasoconstriction during anaphylactic hypotension in anesthetized BALB/c mice. *Exp Lung Res* **39**, 119-129 (2013).

**Figure legends**

**Figure. S1. Tracing presynaptic terminals of dorsal horn neurons that receive kidney afferent input in different brain areas**

**a and b** Schematic for tracing terminals of dorsal horn neurons that receive kidney afferent input (**a**). Terminals of these dorsal horn neurons in different brain areas (**b**). Scale bar, 100 µm. **c and d** Schematic for labeling terminals of SFO neurons that receive kidney spinal afferent input (**c**). Representative image shows terminals of these SFO neurons in MnPO, OVLT and SON (**d**). n=3 in each experiment.

Abbreviations for brain regions: DMV, dorsal motor nucleus of the vagus (Bregma -7.48 mm); NTS, nucleus of the solitary tract (Bregma -7.48 mm); AP, area postrema (Bregma -7.48 mm); PV, paraventricular thalamic nucleus (Bregma -0.94 mm); PVN, paraventricular nucleus (Bregma -0.94 mm); SON, supraoptic nucleus (Bregma -0.82 mm); OVLT, organum vasculosum lamina terminalis (Bregma 0.50 mm); MnPO, median preoptic nucleus (Bregma 0.14 mm); ARC, arcuate nucleus (Bregma -1.46 mm); CeA, central nucleus of the amygdala (Bregma -0.94 mm); LH, lateral hypothalamic area (Bregma -0.94 mm); BnST, bed nucleus of the stria terminalis (Bregma 0.14 mm); AP, area postrema (Bregma -7.48 mm); SFO, subfornical organ (Bregma -0.70 mm); RVLM, rostral ventrolateral medulla (Bregma -6.72 mm).

**Figure. S2. Increase in kidney afferent input promotes progressive cardiac or kidney dysfunction in kidney or myocardial IRI mice**

Selective kidney deafferentation by capsaicin (Cap) or surgical ablation of kidney nerves (KDN) was performed in mice on day 10 after kidney IRI (KIRI), myocardial IRI (MIRI) or sham operation. **a** Kidney expression of calcitonin gene-related peptide (CGRP). Scale bar, 100 µm. **b** Kidney expression of collagen (Co) I protein in KIRI mice. **c** Kidney mRNA level of Co I and fibronectin (FN) in KIRI mice. **d** Glomerular filtration rate determined by inulin clearance in KIRI mice. **e** Cardiac expression of Co I protein in MIRI mice. **f** Myocardial fibrosis determined by Masson staining in MIRI mice: representative images and quantitative data. Scale bar, 100 µm. **g-j** Central venous pressure (CVP, **g**), kidney Ang II concentration (**h**), density of kidney microvessels indicated by endothelial marker CD31 (**i**), and kidney mRNA level of TNF-α, IL-1β and IL-6 in MIRI mice (**j**). Scale bar, 100 µm. **k** Retrograde labeling of SFO neurons projecting to PVN by injecting AAV2/retro-CaMKIIα-EGFP into the PVN. Representative image of injection site in the PVN. Scale bar, 100 µm. **l** Retrograde labeling of PVN neurons projecting to RVLM by injecting CTb-488 into the RVLM. Representative image of injection site in the RVLM. Scale bar, 50 µm. *, *P*<0.001. ns, not significant. Error bars, mean ± SD (n=6 in each group). One-way ANOVA or *t* test with Bonferroni correction.

**Figure. S3.** **Optical silencing of kidney-SFO projection reduces the sympathetic outflow markedly in kidney or myocardial IRI mice**

Optogenetic experiments are performed to silence kidney afferent nerves projecting to SFO. **a** Changes of cardiac SNA in KIRI mice treated with PBS, with (Opt ON) or without (Opt OFF) optical silencing. Scale bar, 2 seconds. The value at 0 minute is set to 0%. *, *P*<0.05 *versus* Opt OFF. **b** Changes of kidney SNA in MIRI mice treated with PBS, with Opt ON or Opt OFF. Scale bar, 2 seconds. The value at 0 minute is set to 0%. *, *P*<0.05 *versus* Opt OFF. Error bars, mean ± SD (n=6 in each group). *t* test.

**Figure. S4.** Selective kidney deafferentation by capsaicin (Cap) reduced dephosphorylation of CRTC1 in SFO of kidney IRI (KIRI, **a**) or myocardial IRI (MIRI, **b**) mice. *, *P*<0.001. ns, not significant. Error bars, mean ± SD (n=6 in each group). One-way ANOVA or *t* test with Bonferroni correction.

**Figure. S5.** **Activation of the SFO RAS promotes progressive cardiac or kidney dysfunction in kidney or myocardial IRI mice**

Deletion of AT1a in SFO is achieved by injection of AAV2/9-Cre into SFO of AT1a*^fl/fl^* mice on day 10 after kidney IRI (KIRI) or myocardial IRI (MIRI). **a and b** Deletion of AT1a in SFO reduces protein level of Co I (**a**) and mRNA level of Co I and FN (**b**) in kidneys from KIRI mice. **c-e** This treatment also decreases myocardial fibrosis (**c**), and reduces protein level of Co I (**d**) and mRNA level of Co I and Co III (**e**) in hearts from MIRI mice. Scale bar, 100 µm. ns, not significant. *, *P*<0.001. Error bars, mean ± SD (n=6 in each group). One-way ANOVA or *t* test with Bonferroni correction.


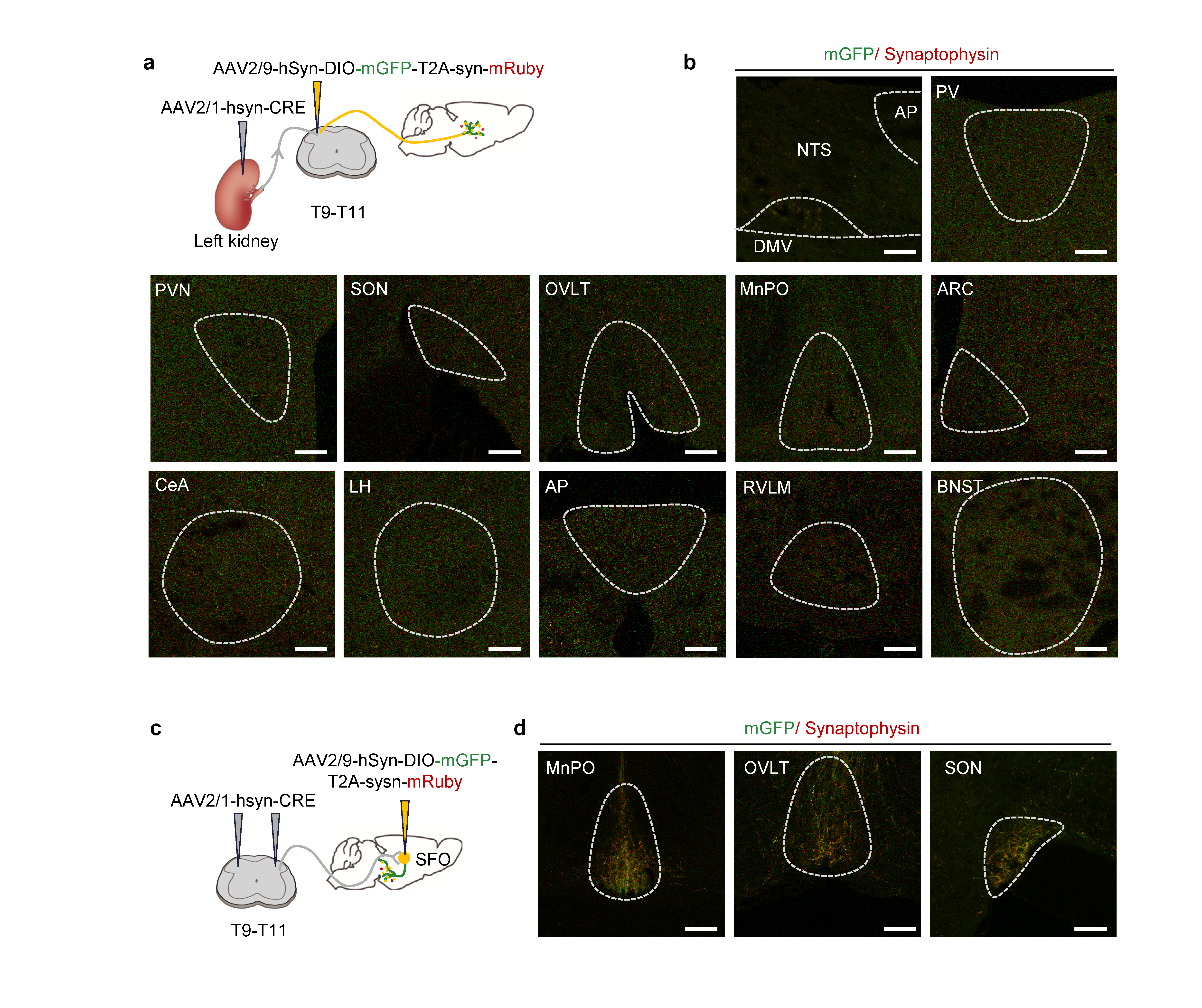


**Figure. S1. Tracing presynaptic terminals of dorsal horn neurons that receive kidney afferent input in different brain areas**

**a and b** Schematic for tracing terminals of dorsal horn neurons that receive kidney afferent input (**a**). Terminals of these dorsal horn neurons in different brain areas (**b**). Scale bar, 100 µm. **c and d** Schematic for labeling terminals of SFO neurons that receive kidney spinal afferent input (**c**). Representative image shows terminals of these SFO neurons in MnPO, OVLT and SON (**d**). n=3 in each experiment.

Abbreviations for brain regions: DMV, dorsal motor nucleus of the vagus (Bregma -7.48 mm); NTS, nucleus of the solitary tract (Bregma -7.48 mm); AP, area postrema (Bregma -7.48 mm); PV, paraventricular thalamic nucleus (Bregma -0.94 mm); PVN, paraventricular nucleus (Bregma -0.94 mm); SON, supraoptic nucleus (Bregma -0.82 mm); OVLT, organum vasculosum lamina terminalis (Bregma 0.50 mm); MnPO, median preoptic nucleus (Bregma 0.14 mm); ARC, arcuate nucleus (Bregma -1.46 mm); CeA, central nucleus of the amygdala (Bregma -0.94 mm); LH, lateral hypothalamic area (Bregma -0.94 mm); BnST, bed nucleus of the stria terminalis (Bregma 0.14 mm); AP, area postrema (Bregma -7.48 mm); SFO, subfornical organ (Bregma -0.70 mm); RVLM, rostral ventrolateral medulla (Bregma -6.72 mm).


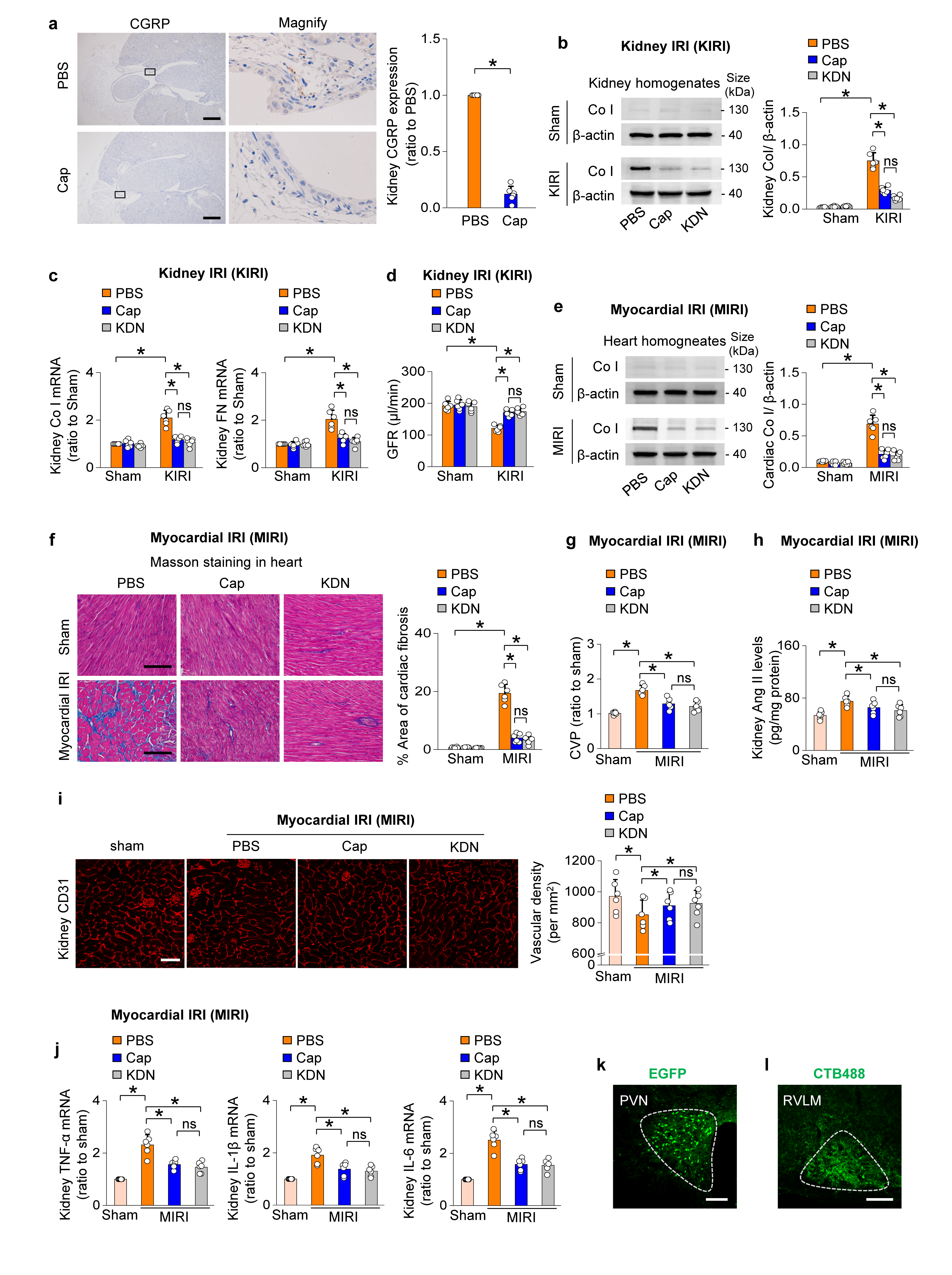


**Figure. S2. Increase in kidney afferent input promotes progressive cardiac or kidney dysfunction in kidney or myocardial IRI mice**

Selective kidney deafferentation by capsaicin (Cap) or surgical ablation of kidney nerves (KDN) was performed in mice on day 10 after kidney IRI (KIRI), myocardial IRI (MIRI) or sham operation. **a** Kidney expression of calcitonin gene-related peptide (CGRP). Scale bar, 100 µm. **b** Kidney expression of collagen (Co) I protein in KIRI mice. **c** Kidney mRNA level of Co I and fibronectin (FN) in KIRI mice. **d** Glomerular filtration rate determined by inulin clearance in KIRI mice. **e** Cardiac expression of Co I protein in MIRI mice. **f** Myocardial fibrosis determined by Masson staining in MIRI mice: representative images and quantitative data. Scale bar, 100 µm. **g-j** Central venous pressure (CVP, **g**), kidney Ang II concentration (**h**), density of kidney microvessels indicated by endothelial marker CD31 (**i**), and kidney mRNA level of TNF-α, IL-1β and IL-6 in MIRI mice (**j**). Scale bar, 100 µm. **k** Retrograde labeling of SFO neurons projecting to PVN by injecting AAV2/retro-CaMKIIα-EGFP into the PVN. Representative image of injection site in the PVN. Scale bar, 100 µm. **l** Retrograde labeling of PVN neurons projecting to RVLM by injecting CTb-488 into the RVLM. Representative image of injection site in the RVLM. Scale bar, 50 µm. *, *P*<0.001. ns, not significant. Error bars, mean ± SD (n=6 in each group). One-way ANOVA or *t* test with Bonferroni correction.


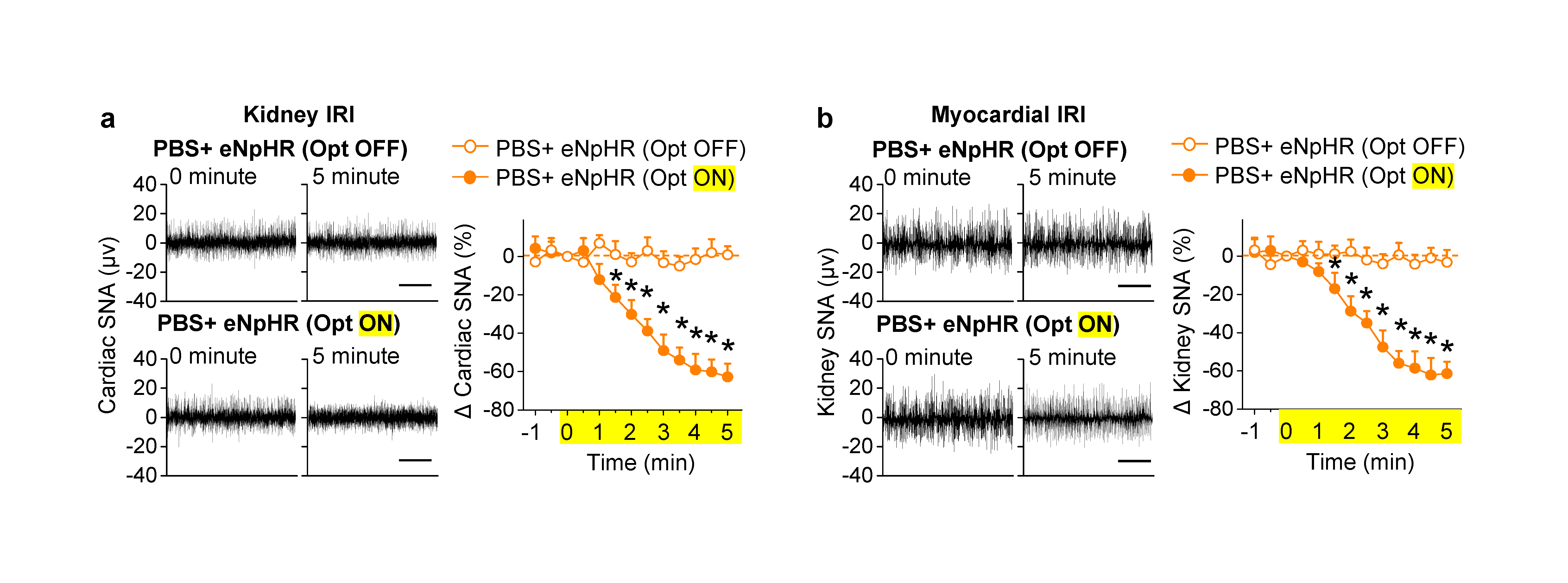


**Figure. S3.** **Optical silencing of kidney-SFO projection reduces the sympathetic outflow markedly in kidney or myocardial IRI mice**

Optogenetic experiments are performed to silence kidney afferent nerves projecting to SFO. **a** Changes of cardiac SNA in KIRI mice treated with PBS, with (Opt ON) or without (Opt OFF) optical silencing. Scale bar, 2 seconds. The value at 0 minute is set to 0%. *, *P*<0.05 *versus* Opt OFF. **b** Changes of kidney SNA in MIRI mice treated with PBS, with Opt ON or Opt OFF. Scale bar, 2 seconds. The value at 0 minute is set to 0%. *, *P*<0.05 *versus* Opt OFF. Error bars, mean ± SD (n=6 in each group). *t* test.


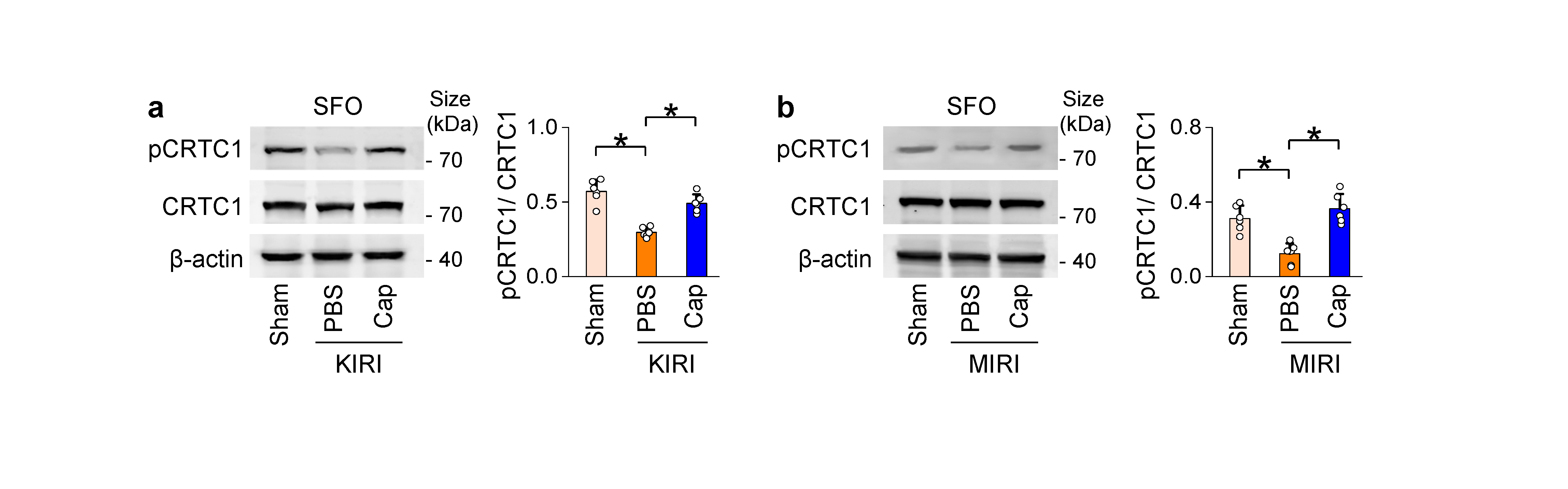


**Figure. S4.** Selective kidney deafferentation by capsaicin (Cap) reduced dephosphorylation of CRTC1 in SFO of kidney IRI (KIRI, **a**) or myocardial IRI (MIRI, **b**) mice. *, *P*<0.001. ns, not significant. Error bars, mean ± SD (n=6 in each group). One-way ANOVA or *t* test with Bonferroni correction.


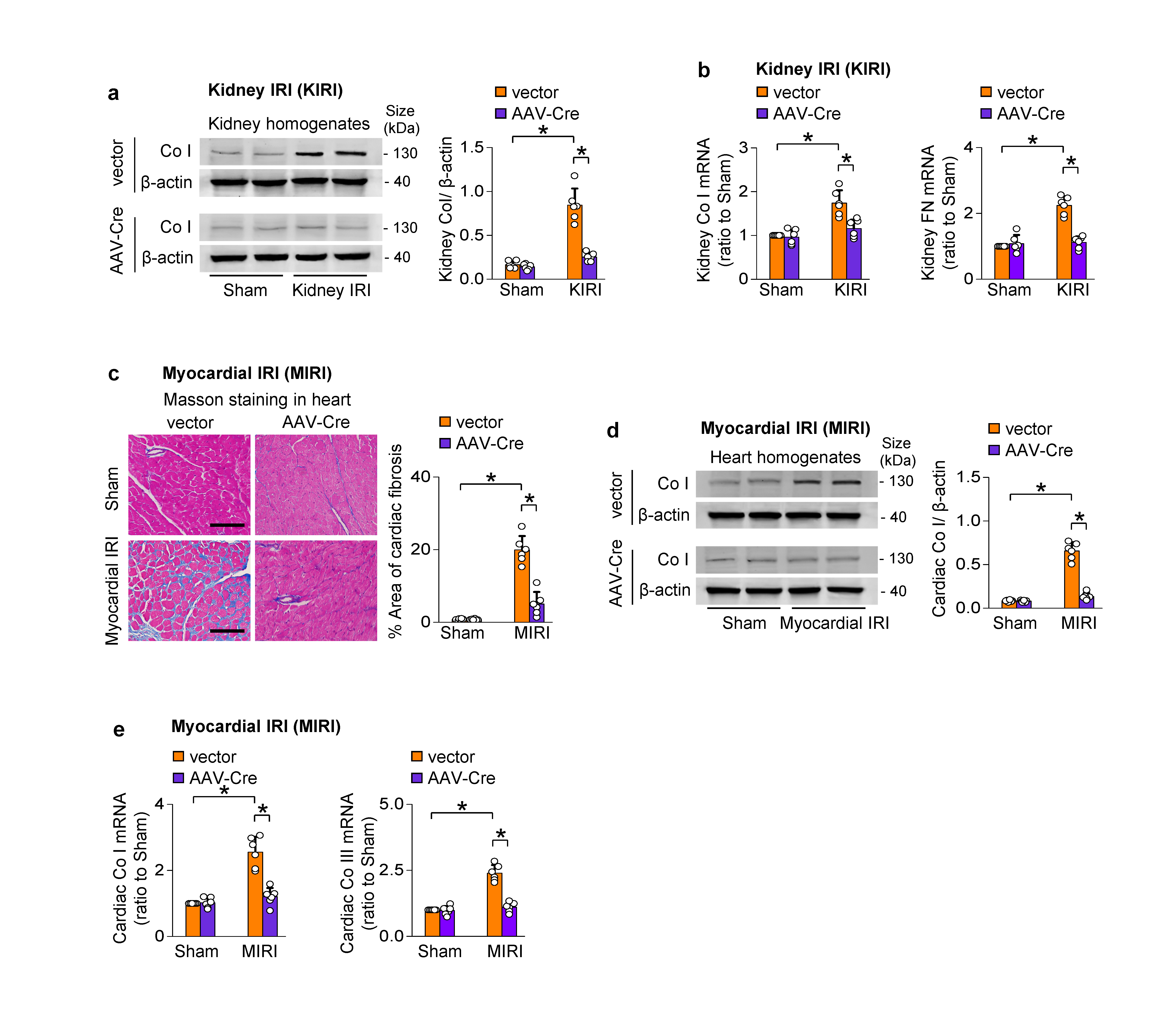


**Figure. S5. Activation of the SFO RAS promotes progressive cardiac or kidney dysfunction in kidney or myocardial IRI mice**

Deletion of AT1a in SFO is achieved by injection of AAV2/9-Cre into SFO of AT1a*^fl/fl^* mice on day 10 after kidney IRI (KIRI) or myocardial IRI (MIRI). **a and b** Deletion of AT1a in SFO reduces protein level of Co I (**a**) and mRNA level of Co I and FN (**b**) in kidneys from KIRI mice. **c-e** This treatment also decreases myocardial fibrosis (**c**), and reduces protein level of Co I (**d**) and mRNA level of Co I and Co III (**e**) in hearts from MIRI mice. Scale bar, 100 µm. ns, not significant. *, *P*<0.001. Error bars, mean ± SD (n=6 in each group). One-way ANOVA or *t* test with Bonferroni correction.

**Table. S1. Primers used for Real-time PCR**

| Gene | Forward (5’->3’) | Reverse (5’->3’) |
| --- | --- | --- |
| *Agtr1a* | TGGGCGTCATCCATGACTGTA | TGAGTGCGACTTGGCCTTTG |
| *Agt* | GGAACGACCTCCTGACTTGG | TCAGATTTGCCTCCGCACC |
| *Col1a1*  *Col3a1*  *Fn*  *TNF-α*  *IL-1β*  *IL-6* | GACAGGCGAACAAGGTGACAGAG  AGAACCTGGCCGAGATG  AGTGGCTGAAGTCGCAAGGAAAC  CATCTTCTCAAAATTCGAGTGACAA  TGCCACCTTTTGACAGTGATG  AAAGAGTTGTGCAATGGCAATTCT | CAGGAGAACCAGGAGAACCAGGAG  TGGACTTCCGGGCATAC  TAAGTCTGGGTCACGGCTGTCTC  TGGGAGTAGACAAGGTACAAACCC  AAGGTCCACGGGAAAGACAC  AAGTGCATCATCGTTGTTCATACA |
| *Gapdh* | GCACAGTCAAGGCCGAGAAT | GCCTTCTCCATGGTGGTGAA |

**Uncropped films of Western blots**


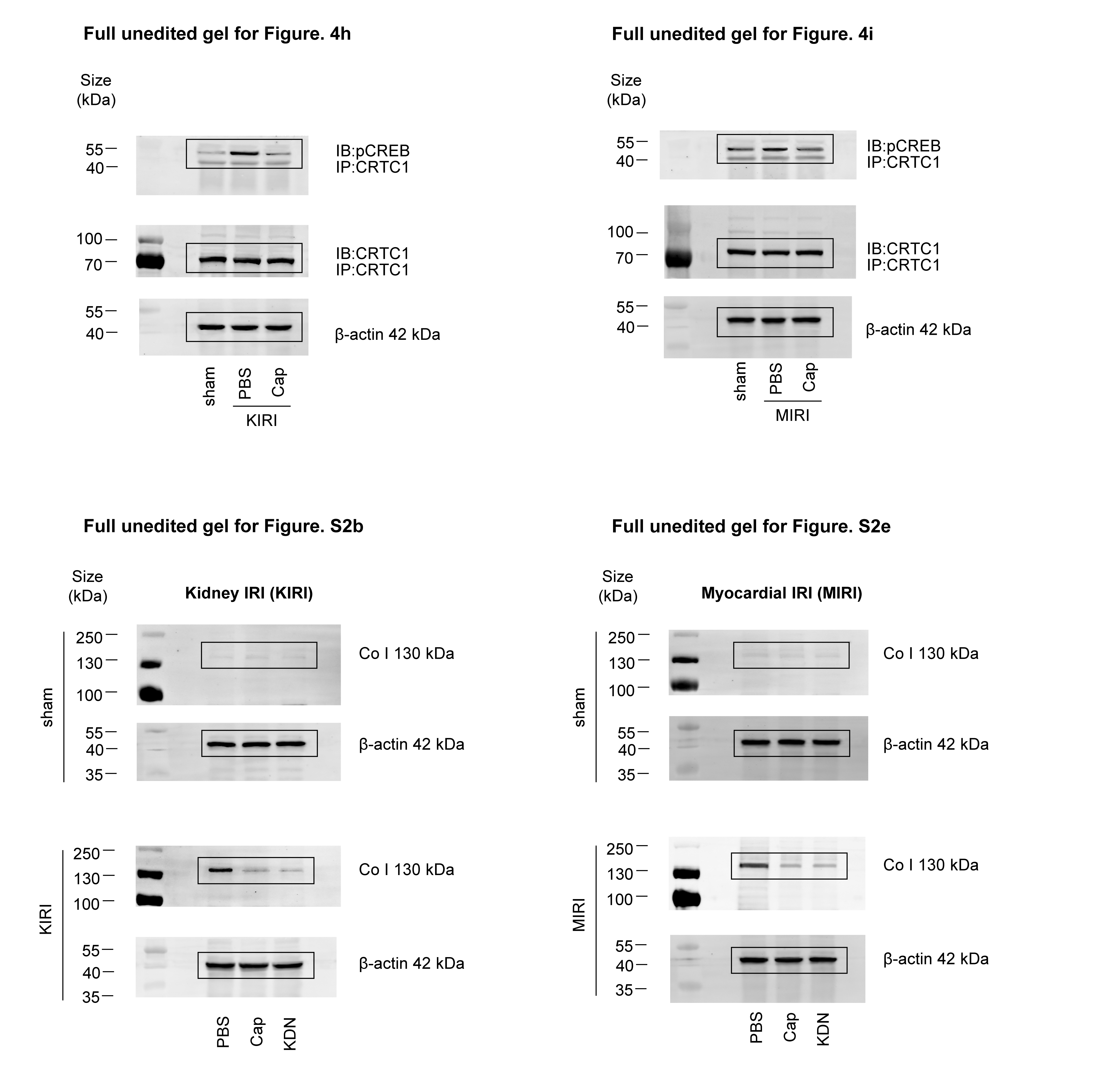


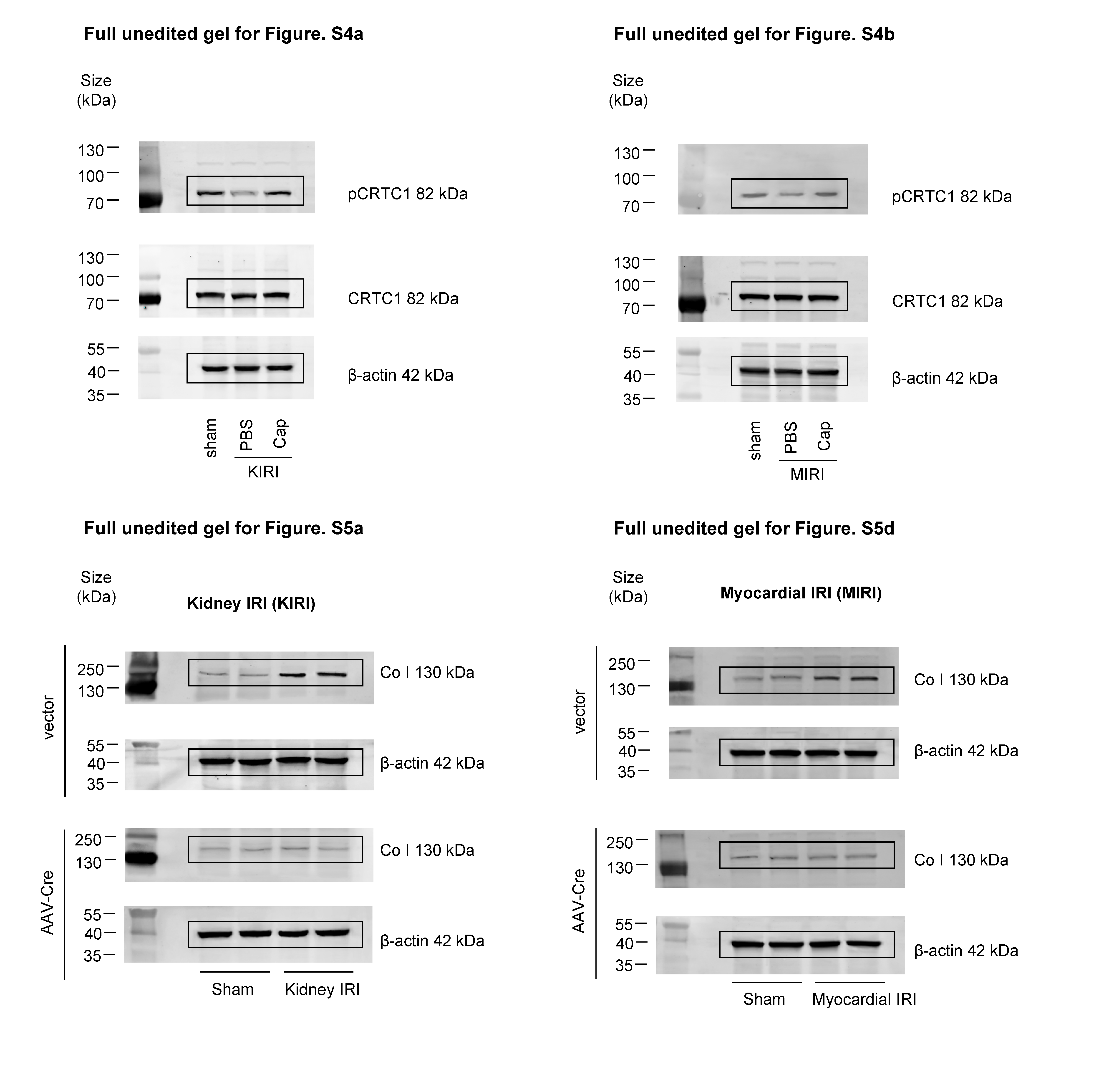

Supplement: Supplementary file 1 — Supplementary Materials [file 41392_2023_1402_MOESM1_ESM.docx]
